# Parental Perspectives of a 4-Week Family-Based Lifestyle Intervention for Children with Obesity

**DOI:** 10.5539/gjhs.v5n2p111

**Published:** 2012-12-17

**Authors:** Erin S. Pearson, Jennifer D. Irwin, Shauna M. Burke, Sheree Shapiro

**Affiliations:** 1 Faculty of Health and Behavioural Sciences, School of Kinesiology, Lakehead University, Thunder Bay, Canada; 2 Faculty of Health Sciences, Western University, London, Canada; 3 Toronto Public Health, Toronto, Canada

**Keywords:** lifestyle program, obesity, child, physical activity, behaviour modification, parent

## Abstract

**Objectives:**

The childhood obesity epidemic is now recognized as one of the most serious public health challenges of the 21^st^ century. Community-based behaviour modification treatment programs involving both children and their families are warranted. The purpose of this study was to explore the experiences of parents whose children participated in the Children's Health and Activity Modification Program (C.H.A.M.P.): a 4-week lifestyle program delivered as a day-camp for obese children at risk for type II diabetes and their families. Parents were required to attend four half-day education sessions during the intervention period.

**Methods:**

Seven focus groups were conducted immediately following the 4-week interventions offered in August 2008 and 2009. The perspectives of 38 parents representing 32 children aged 8-14 with obesity (i.e., body mass index > the 95^th^ percentile) were shared.

**Results:**

Overall, parents were pleased with the impact of the program and proud of their children's accomplishments (e.g., increased physical activity levels, enhanced self-esteem, weight loss). Several facilitators to success (e.g., social support; a positive environment) and barriers to its maintenance (e.g., time management; unsupportive family members) were identified, and recommendations were made for future programs. Although parents found the half-day sessions valuable, post-programmatic bi-monthly booster session adherence declined over the one-year follow-up period.

**Conclusion:**

Delivered as a 4-week day-camp, C.H.A.M.P. represents a unique approach to the treatment of childhood obesity. Future family-based interventions should consider avenues for intensifying the parental program component whilst employing strategies to promote parental adherence in service of enhancing long-term sustainability of health behaviour changes.

## 1. Introduction

The childhood obesity epidemic is now recognized as one of the most serious public health challenges of the 21^st^ century (World Health Organization, 2011). In Canada, recent data indicate that 26% of children are either overweight or obese (Shields, 2006). Not only are these rates alarming, but they have serious implications given that obesity among children is maintained often into adulthood (Deckelbaum & Williams, 2001; Whitaker, Wright, Pepe, Seidel, & Dietz, 1997). Beyond the physical health risks associated with childhood obesity (e.g., type II diabetes, hypertension, advanced growth and early maturity; Summerbell et al., 2003), when compared to their normal weight counterparts, these children are also more prone to stigma experiences (Puhl & Brownell, 2001) and the development of psychological manifestations such as low self-esteem and depression (Israel & Ivanova, 2002; Needham & Crosnoe, 2005; Strauss, 2000). Given that early life events play an integral role in shaping an individual's health trajectory (U.S. Department of Health and Human Services [USDHHS], 2010), it is vital to intervene during childhood, a time when many contributing lifestyle factors and behaviours are amenable to change.

The broader community environment has been shown to affect strongly, an individual's capacity to be healthy as a function of biologic, physical, and social influences (USDHHS, 2010). For health interventions aimed at changing individual behaviour, inclusion of a whole-person, whole family, and whole community approach has been recommended (USDHHS, 2010). The Children's Health and Activity Modification Program (C.H.A.M.P.), a 4-week pilot lifestyle intervention delivered as a summer day-camp from Monday to Friday during August of 2008 and 2009, was developed in response to a paucity of community-based research for childhood obesity targeting both children and parents (Summerbell et al., 2003). Previous behavioural modification interventions have shown that the direct involvement of at least one parent during treatment improves short- and long-term weight regulation (Berry et al., 2004; Epstein, 1996; Epstein, Paluch, Roemmich, & Beecher, 2007). Informed by recommendations advanced by the American Dietetic Association (2008) along with the use of several evidence-based group dynamics strategies (Martin et al., 2009), the curriculum for the children featured a variety of daily physical activities (i.e., aerobic, resistance, and flexibility activities) for at least four hours per day, as well as educational sessions focusing on self-regulation and behaviour modification strategies (e.g., related to nutrition, self-esteem, and physical activity). Parents were required to attend similarly themed half-day educational sessions for four consecutive Saturdays during August. Invited speakers presented to the parents on topics such as healthy and positive child-rearing skills (e.g., how to be supportive of change, acknowledging feelings, and tapping into values); nutrition (e.g., label reading, serving sizes, and psychological aspects of eating); bullying; goal setting; and self-esteem building. Post-program support was offered to families in the form of five, 2-hour booster sessions which were held bi-monthly for one year following the program. These group-based “refresher sessions” were led by the researchers and/or other health professionals, and included an overview of camp curricula (in addition to new health-related information and resources), as well as opportunities for parents and children to participate in physical activity as a group (for a complete description of C.H.A.M.P., please refer to Martin et al., 2009).

Results obtained through a feasibility analysis of the program using the RE-AIM framework, an evaluation tool intended for community-based health interventions (Glasgow, Vogt, & Boles, 1999), revealed significant improvements from baseline to post-intervention (i.e., a four week duration) among the children for body composition and psychological measures (Shapiro et al., under review). For example, significant decreases were observed from pre- to post-intervention for standardized body mass index (BMI-*z*) and percentage of fat mass, while percentage of muscle mass increased significantly. Health-related quality of life (QoL) was assessed using the Pediatric Quality of Life Inventory 4.0 (Peds-QL 4.0; Varni, Seid, & Rode, 1999), which includes a proxy measure of parents’ perceptions of their child's *physical*, *emotional, and social* health-related QoL. Insofar as the parents’ perceptions were concerned, significant improvements were observed for all three types of QoL from baseline to post-intervention, and all were associated with a large effect size (i.e., *physical*, η^2^ = 0.26; *emotional*, η^2^ = 0.18; and *social*, η^2^ = 0.32; Shapiro et al., under review).

To our knowledge, C.H.A.M.P. was the first program of its kind in Canada; thus, examining the viewpoints of participants was deemed an essential complement to this quantitative assessment in order to inform future research involving this at-risk population. Therefore, the purpose of the present study was to explore the perspectives of the parents whose children participated in C.H.A.M.P. with a view toward uncovering barriers or facilitators for healthy behaviour change for obese children and their families. A secondary purpose was to identify impactful program components that could be considered useful for prospective treatments while examining parental adherence during and following the formal intervention up to one year.

## 2. Methods

Based on their availability, parents signed up to attend one of seven focus groups held on either a Saturday morning or weeknight during September of 2008 and 2009. No restrictions were made pertaining to the number of parents allowed to represent one child. Each 60-90 minute recorded session was facilitated by a moderator and moderator-assistant trained in focus group methodology, and followed a semi-structured interview guide to ensure consistency across groups while allowing for flexibility in responses (see Table 1). After signing a consent form, participants were asked about their experiences as a C.H.A.M.P. parent, the impact of the program on their family, and their perceptions of their child's experiences. Questions were developed based on tenets ascribed to social cognitive theory (e.g., self-efficacy; Bandura, 1989). Specifically, participants were asked about their child's experiences pertaining to the primary programmatic components of the intervention including: a) physical activity and nutrition; b) what it was like to be part of a team; and c) how they felt about themselves (i.e., self-esteem). Once the sessions were transcribed verbatim, inductive content analysis (Miller & Crabtree, 1999) was used to code and categorize the data by three researchers independently, who later met to discuss and corroborate their findings. Intercoder reliability was evaluated using reliability checks throughout the data analysis period (i.e., reviewing disconfirming evidence and debriefing; Cresswell & Plano Clark, 2007). In the event that a discrepancy arose between the researchers regarding differential interpretations of the data, discussion ensued until consensus was reached and refinements were made to improve consistency. In accordance with Lincoln and Guba (1985) and using specific methods described previously (Irwin, He, Sangster Bouck, Tucker, & Pollett, 2005), multiple strategies were implemented throughout the study to ensure data trustworthiness (i.e., credibility, dependability, confirmability, and transferability). Ethical approval was obtained through the University's research ethics board.

**Table 1:** Abbreviated semi-structured focus group guide for parents

**PART I: Parental Perceptions of CHAMP**
**Saturday Sessions:** The first few questions I am going to ask you will focus on the Saturday education sessions.
Aside from nutrition (which we will talk about in a moment), what did you find most valuable from the Saturday sessions? What did you find the least helpful? Why?
What, if anything, is different about parenting for you now since attending CHAMP?
How does what you’ve learned at CHAMP about health and parenting make you feel? (i.e., ability, confidence, knowledge)
How do you use what you have learned during these family sessions at home?
• Probe: How have your families responded?
**Nutrition:** The next few questions will focus on the nutrition portion of the CHAMP Saturday sessions.
What did you find most helpful/useful about the nutrition part of the program? Why? What did you find the least helpful? Why?
In what ways do you use what you’ve learned about nutrition at home?
• Probe: How have your families responded?
Self-Esteem: For the next set of questions, I would like you to think about how CHAMP has influenced that way that you feel and act towards yourself and your family.
Since coming to CHAMP, what (if anything) is different about how you treat yourself? Your family?
• Probe: Could have to do with physical activity, healthy eating
What have you learned about yourself? About your family?
**Cohesion:** The following questions focus on your relationships with the other CHAMP parents and guardians.
In what ways did you interact with other parents or families during CHAMP?
• Probe: Was this helpful? Why or why not?
What kind of contact (if any) have you had with the other CHAMP families since CHAMP ended?
• Probe: How often? What types of things did you do/discuss?
**General:**
What would you say is the single most important thing that you learned at CHAMP?
How are you going to incorporate what you’ve learned at CHAMP into your family life now that CHAMP is finished?
What (if anything) would you like to see more of in future CHAMP parent education sessions? Less of?

**PART II: Parental Perception of the Impact on their Children**
Now I am going to ask you some questions about your children and how you think CHAMP has impacted them.
**Physical Activity:**
What is different about physical activity for your child now since coming to CHAMP?
• Probe: What have you noticed about how your child views physical activity now?
• Probe: How do you think your child will manage to sustain his or her new behaviours in the home environment now that CHAMP is finished?
How do you think he or she feels about being more physically active?
• Probe: What would you say that he/she liked best about the physical activity part of the program (i.e., activities? Least?)
**Nutrition:**
What is different about nutrition and eating for your child now since coming to CHAMP?
In what ways has your child applied what he/she has learned about nutrition at home?
• Probe: How do you think your child will manage to sustain his or her new eating behaviours in the home environment now that CHAMP is finished?
In what ways has your child applied what he/she has learned about nutrition at school?
In what ways (if any) do you as a family plan to eat differently at home now that CHAMP is finished?
**Self-Esteem:**
What is different about how your child feels about him/herself since coming to CHAMP?
• Probe: Can you give an example of that? What is different about how your child treats him/herself since coming to CHAMP?
• Probe: Can you give an example of that? What has your child learned about him/herself?
**Cohesion:**
What types of supports and/or barriers do you feel the children provided one another during CHAMP?
• Probe: Do you anticipate that these will continue now that CHAMP is finished?
In what ways do you think that being part of a team and participating in team games at CHAMP impacted your child?
• Probe: How did being involved in the CHAMP “group” make your child feel?
What kind of contact (if any) did your child have with other CHAMP campers outside of camp while CHAMP was running?
• Probe: Do you feel that it is important for the children to have contact with other campers outside of CHAMP?
What kind of contact does your child have with other CHAMP campers now? What types of things do they do?
LAST QUESTION: At the start of CHAMP, how did you feel about being involved in a program targeting overweight children?
How do you think your child felt?
• Did these feelings remain the same, or did they change over the course of the program? In what ways?
• Is there anything else about CHAMP that we haven’t discussed that you would like to add?

## 3. Results

The perspectives of 38 parents (mothers = 30; fathers/step-fathers = 8) representing 32 children aged 8-14 with obesity (i.e., body mass index > the 95^th^ percentile for age and sex) were shared following the camp-based portion of this two year project (2008 = 3 groups [*n* = 6; *n* = 6, *n* = 5]; 2009 = 4 groups [*n* = 7; *n* = 8; *n* = 3; *n* = 3]). Participant demographics for the parents were collected via questionnaire administration which occurred in July during the baseline assessment for each participant (see Table 2).

**Table 2:** Parental demographics

Participants (*n*=38 unless indicated otherwise)	Percentage (%)
Female	79
Male	21
Annual family income per household (*n*=27)*	
less than $50,000	15
$50,000 to less than $60,000	22
$60,000 to less than $80,000	7
$70,000 to less than $90,000	26
$80,000 or more	30
Highest education level	
Some or completed high school	13
Some or completed college	68
Some or completed university	16
Graduate degree	3
Current employment	
Part time	8
Employed	87
No paid employment	5
Ethnicity	
Caucasian	97
Other	3

* Note: ‘*n*’ is based on the number of respondents to this particular question.

The mean attendance rates for parents involved in the family sessions from Weeks 1 through 4 were as follows: 2008 = 73.3%, 46.7%, 60.0%, and 80.0%; 2009 = 70.8%, 60.4%, 62.5%, and 100.0%. Analysis of post-program adherence among parents attending the bi-monthly booster sessions which were held for one year following the formal intervention (*n* = 5) revealed a decline in attendance rates for both years of the program (2008: 60.0%, 53.3%, 21.4%, 28.6%, and 28.6%; 2009: 66.6%, 41.7%, 41.7%, 25.0%, and 16.7%).

Five overarching themes and 10 subthemes emerged from the focus group discussions. These included: parental changes attributed to the program (i.e., increased awareness and modelling; empowerment and trust); the program's psychosocial impact on the children (i.e., improved self-esteem and enhanced self-efficacy); facilitators for change (i.e., camp structure and culture; specific tools and skills; social support); barriers to change (i.e., time management; family members); and recommendations for future research (i.e., more education and opportunities; enhanced accountability). Descriptions and examples pertaining to each are detailed below.

### 3.1 Parental Changes Attributed to C.H.A.M.P.

#### 3.1.1 Increased Awareness and Modelling

Overall, parents expressed that that they were much more aware of the risks and benefits associated with their lifestyle choices and the importance of integrating intervention learnings into the daily routines of their families. Many described a new or heightened awareness of how their own thoughts and behaviours influenced those of their children; they described frequently the importance of leading by example, acting as a role model, and the realization that “it starts with you”. Less consistently but intensely voiced was an increased sense of self-acceptance among parents (e.g., not being as hard on themselves), especially with regard to deserving time for, and becoming healthy themselves. Quotations pertaining to increased awareness and modelling are displayed in Table 3.

**Table 3:** Parental changes attributed to the C.H.A.M.P. program

Increased awareness and modelling
• “This whole CHAMP thing has been good for me personally, and the family, because it…reminds you what’s important- the sodium levels, the sugar levels…I really enjoyed the diabetes [education] because it was a really good, scary reminder of what can happen if you don’t do stuff….”
• “Because I know what they [my children] look like…and that they’re heading towards where I am, and I don’t want that. So if I can do this now…if I can get me to feel and look better, then I think it will really help them too. I really do.”
• “I’d say I’m more conscious about me. Just how I’m taking care of me and taking that time for me…Because they [my children] see it.”
• “[I realize] [j]ust how important it is for me to keep my word or my end of the bargain…I mean, kids are a product of their environment…in order for [my daughter] to get to where she needs to be, she needs to be able to follow my husband and myself so she knows how to get there. So it’s…being really aware of that, I think is the most important part for me.”
• “[I]t has to be a family… thing. Like…you can’t just have the kid go and…expect them to make the change. I mean,…we have to reinforce it and I think we have to want it for them. But we have to also want it for ourselves in order to…make it work.”

Empowerment and trust
• “Right now, it’s just, we’re more empowered to go and take those steps…and say ‘No, you know better. I know you know better, and I know I know better.’”
• “Empowered. Yeah, definitely. You feel like you have more knowledge to…work everything out, you know? Any of that. Whether it’s with the nutrition or exercise … You feel like you have some more tools to kind of deal with things.”
• “I know that, personally, I’m accepting more of our son’s feedback. I think I am more confident that he understands better when we talk about nutrition…exercise and health…I’m more confident in his understanding of it, so I, I give more credence to what he says.”
• “We [as parents] are listening a lot more, and…interacting more, just sitting and talking to him [my son] more, like a person and… not quite an equal, but not as just a child either…because he did take a lot more away from this [C.H.A.M.P.] than what I ever expected he would, and he retained so much more than we ever thought he would. So when we do talk, it’s just like talking to another person.”
• “I think the way we go about it is different now. Before it was always, you know, ‘If you keep eating like that….you’re gonna get bigger and bigger.’ But now, because he can relate to portion size and nutrition…he knows what’s good for him and he knows what his ‘sometimes’ foods are…but we can talk almost the same language. Whereas before, we would talk and he would have to listen, but now he knows what we’re talking about.”
• “I think [my daughter] realizes now she has the tools to do things, but it’s up to her to…That mom and dad don’t always have to say ‘You have to do this and that.’ That you can indeed take responsibility for yourself and it’s up to you to do that.”
• “[Now], they [the kids at C.H.A.M.P.] actually understand what’s going on, and whether it’s physical activity or…it’s like they have a sense of understanding; what you should be doing, even if, you know, they might not want to do it…they get something that’s important. They didn’t get that before.”

#### 3.1.2 Empowerment and Trust

When asked what changes in parenting had occurred since attending C.H.A.M.P., participants commonly alluded to a parent-child relationship shift involving new-found empowerment and trust experienced by both the parent and child. Specifically, parents stated repeatedly that they felt “empowered” to help their children as a result of the information they received. This involved their feeling more in-control and confident with respect to decision-making and parenting skills, and better equipped with the knowledge and tools to implement healthy changes. In a similar vein, parents reported “empowering” the children more and trusting that they would make healthy decisions (e.g., allowing them to serve themselves at a meal and make their own lunches). Related to this shift, parents also expressed that they had a different appreciation of the parent-child relationship, a more positive outlook, and were communicating with their children more effectively (e.g., they felt like they were nagging less and having more interactive conversations). Table 3 provides depictive comments.

### 3.2 The Program's Psychosocial Impact on the Children

*Improved Self-esteem and Enhanced Self-efficacy*. Across all discussions, parents reported seeing improvements in the self-esteem levels of the children. Specifically, parents noted that the children were: taking more pride in how they looked; increasingly positive in their self-talk and attitudes; more comfortable with being themselves; and proud of themselves and their accomplishments (e.g., wearing a smaller clothing size).

Related to these improvements in self-esteem, parents indicated that the children were: increasingly confident in their ability to perform particular behaviours (e.g., running), more capable and willing to try new things (e.g., activities, sports, or foods), and able to build physical endurance and enjoy activities more as a result. Parents pointed out that the children were now taking initiative by applying the skills and knowledge that they obtained through the program, and also suggested that the children wanted to share what they were learning with friends and family members. Parental accounts of the program's impact on the children can be found in Table 4.

**Table 4:** The program’s psychosocial impact on the children

Increased self-esteem and enhanced self-efficacy
• “My son is very proud of himself and his accomplishments and shares it with absolutely everybody!…I don’t think he ever thought that he could do it, and I don’t think he ever thought he could do it and enjoy it.”
• “[My daughter] didn’t lose any pounds…but she knows that going back to school…all those clothes that she could not fit into that she loved…She’s lost that little stomach; she’s built muscle… she’s back into all her clothes and she’s over the moon about it!”
• “[My daughter] will accept a compliment. Like, instead of just [crying]…brushing it off or being like ‘yeah whatever. ’ But it’s like, ‘Thanks!’ [that’s different for her]…I almost fell over…[I learned that] she’s capable of a lot more and that she wants a lot more. I don’t think she realized how much she was missing, going out and playing with other kids and so I think that she came to that realization that she wants to do things.”
• “I think [my daughter] cares more about her appearance now. And before she just, whatever, she’d just get dressed. She didn’t care if her clothes matched, ‘cause half the time we couldn’t find clothes that were nice stylish clothes anyway. But now, she’s…really noticing and she wants to go shopping…”
• “Yeah, it’s the confidence… it’s the energy level. Like I said, [my son] runs everywhere now. And then the tests [intervention assessments] help, because it re-iterated…when we did all the scans and saw the difference. Like you could tell…I can see it in his body… [and] he’s noticed it…So he’s healthier and I can tell that. He has more energy and so goes from A to B a lot faster.”
• “The confidence that they can do things, try something new and not be afraid and to just, to you know, that you’ve empowered them and given them the self-confidence to just try it.
• “[I learned that my daughter is] stronger than what she gave herself credit for…and willing to take the risk [to try new things].”

### 3.3 Facilitators for Change

#### 3.3.1 Camp Structure and Culture

Parents described several C.H.A.M.P. features to which they attributed some of the positive changes that were experienced. A wide variety of physical activities, supportive staff who pushed the kids in an encouraging manner, the camp location (i.e., somewhere “safe” that was away from other sport and summer camps), and a non-judgmental environment were all cited frequently. Many parents described how the children held information received from the counsellors in much higher regard than if it had come from the parents themselves, and that this was an important facilitator for their learning. Parents commented that the camp focus on overall health and participation (versus results and weight-loss) was beneficial for the children. They also stated that they appreciated receiving the same information on weekends that the children received during the week (e.g., pertaining to nutrition and physical activity); this enabled them to speak the same language, reinforce concepts at home, and hold each other accountable for changes. Finally, many parents expressed that the cost of the camp itself ($200.00 for 4-weeks, not including lunches; Martin et al., 2009) facilitated their decision to enroll.^1^

#### 3.3.2 Specific Tools and Skills

As discussed previously, parents were provided with information, resources, and tools during the family-based component of the program. Parents identified several of the tools and skills that were provided and emphasized in these sessions as helpful in working with their family members to make healthy changes. Visual tools were mentioned consistently, and included: engaging in goal setting as a family and posting action plans around the house; working with food models illustrating portion sizes, fat, and sugar content of commonly enjoyed foods; and using hand-outs containing suggestions for lunches and sample meal plans. Parents reported that they found the grocery store tour valuable (part of the parental curricula) as it provided hands-on experience and practical information pertaining to food content, portion sizes, and marketing strategies. Making a conscious effort to plan ahead (e.g., preparing meals on Sunday for the week; arranging family time to do physical activity) was also mentioned frequently.

#### 3.3.3 Social Support

Across all focus groups, parents expressed that they felt comforted knowing that there were other families who faced the same challenges that they were experiencing. There was also a widespread view that the children felt less isolated, more accepted, and like they “just fit in” as a result of participating in the camp. Parents attributed these feelings, in large part, to the fact that the children did not feel as though they were being judged by anyone at camp, and noted that through their C.H.A.M.P. teams (Martin et al., 2009), the children were able to encourage and motivate one another (see Table 5).

**Table 5:** Facilitators and barriers for change

Facilitators
• “[T]he focus was on effort and participation not on results, whereas a lot of camps are results-oriented. So it was nice that they were all encouraging, and really supportive, and applauding best efforts. It was nurturing in that regard as well.”
• “Having other kids there that were trying things new for the very first time… so she knew nobody was judging her and that was key. Nobody was judging her on how well she was doing and… she knew that there wouldn’t be a child who just accelerated above all the rest; she knew that they were all in the same level…So that in itself gave her the empowerment to…try new things.”
• “I learned that other people have way more influence over [my child] than I do… My mom asked me how it went, and I said, ‘Mom, for once [there was] a counselor for every two kids…It was the most positive experience ever. Everybody was all for them, and I don’t think everyone ever gets that in their lives.’”
• “Well, I think the first day…they were sold right from the get go…Just the acceptance… everything was so positive for them and it was all about them and they liked everyone. And I think they felt genuinely liked as well. In other words no opinions, nothing negative, no judgement.”
• “[My child] and I did it [goal setting]…I find we need to have it in writing. And it’s up on our wall, in between our two bedrooms, and we need to keep looking at it and reminding ourselves because we’re so busy now that summer’s over; work, school, homework. So it helps us.…”
• “I feel like I’m not alone. And it’s a nice feeling to know that there are so many other people out there just like me…We’re really feeling comfortable as a group and I got the feeling… that I can do it. It’s not just me.”
• “[My son] just seems to have a little more, self-assured. I think the camp and the kids at the camp gave that to him…It was just being in the group…I remember the first week he said to me: ‘Mom, everybody belongs…I just know that I’ll fit in.’”
• “There was so much positive reinforcement. Like it was always high fives and ‘way to go’ or whatever.”
• “Ya, let’s face it. At school, he’s the outsider…This was his place to go, some place, and be welcomed in and … get the positive feedback for … trying hard at this and doing that. I think that was…that was huge for him.”
• “Some goals we put in writing, and I found that’s the only way actually to get anything done.”
• “Here is a program that…can keep the price real…they say that it’s, you know, people who come from lower income who have these issues…right? So, it was really nice to see that they kept the price of it really realistic, so that there’s finally something out there that can help, where you don’t have to…spend the farm on it to get these kind of results and this kind of knowledge and education. That was really cool.”

Barriers
• “That’s the barrier. It’s time. It’s getting home, and you’re not getting home ‘til 5:30, then you’re eating dinner and then you got to turn around and get going and go out somewhere else….”
• “I’m struggling. I have a real barrier. I know I’m being a barrier because she wants me to come on and do everything with her, and I can’t… So, how do you get past that barrier, you know?
• “Well, I still think it’s going to be a struggle…if it’s not structured for him, he still likes to watch TV or go on the computer. And so I’m going to have to try scheduling something for him to do…I’ve asked him if he wants to do certain things already for the fall and his answer is no. He doesn’t want to do those things that I’ve asked him.”
• “[W]e have no buy-in from [our older son]. I mean, he’s the one who eats nothing but garbage and is you know, half the size of [our younger son]…When [our younger son] was a little kid he would say…‘How come [my older brother] eats so much crappy food and he’s so skinny and I’m so fat?.”
• “I feel a bit lost…we have a split relationship with the children. One week at our house, one week at their father’s house. And [the father has] only come to one session…he didn’t really care about this….”

### 3.4 Barriers to Change

#### 3.4.1 Time Management

Many parents expressed that making healthy changes was challenging at times due to busy schedules. Interestingly, some parents identified themselves as a barrier to making healthy changes for their family members due to challenges with scheduling and feeling fatigued at the end of the day.

#### 3.4.2 Family Members

Frustration was also expressed by parents who felt that their family members were not supporting the changes they were trying to implement. Siblings were identified commonly as a barrier and this was especially the case when they were not struggling with overweight themselves. Quotations pertaining to barriers are displayed in Table 5.

### 3.5 Recommendations for Future Programs Involving Children with Obesity and Their Families

#### 3.5.1 More Education and Opportunities

Despite some concerns about the timing of the parent sessions (i.e., every Saturday during August), what was voiced consistently and strongly across all groups was the parents’ desire for more: in depth information and tips; frequently held sessions of longer duration; time with the professionals and guest presenters; hands-on learning opportunities; take-home materials; and ideas for meals. Beyond the program content, parents also voiced the need for more social networking opportunities during the program to share with and learn from other parents. The desire for more personalized feedback and opportunities to spend time at camp with the children were also mentioned.

#### 3.5.2 Enhanced Accountability

Some parents conveyed that they wanted to be held more accountable by program staff for their “homework assignments” and lifestyle change attempts. Recommendations for future programming included making parents more aware at the outset of camp, of the role that they need to play for their family members, and having staff members check-in frequently to ensure that families are following through with their goal setting. Concerns about the future and what would happen now that the camp was over were also reported. Quotations illustrating recommendations for future research can be found in Table 6.

**Table 6:** Recommendations for future programs

• “I would have preferred to have done all of it [attending camp] with him…I think we would have benefited doing it, the family sessions as a family together. And also, the opportunity to spend a day at camp, because he always goes to me, ‘You have no idea, you should come to camp!’ …There should be a CHAMP camp for parents!”
• “I think it would have been really nice to [have] maybe met the [other] parents more…just so we could have gotten to know each other because I think that may have helped with continuing communication…afterwards.”
• “I thought it would be neat if even for an hour during the month, if the parents could go and do something with the kids [at the camp]. Like, I’d love to see the whole class in action when they’re…all on their bikes doing their thing.”
• “Only to be made more accountable… I looked at it afterwards [sigh]…Everyday they bring something home, and yea, we’re supposed to sign this and look at this. I didn’t do that to the extent I should have.”
• “I wish that there had been a more practical…examples of what you can do for lunch. Lunches are a big thing. Every day, it’s an apple, it’s a sandwich…And now you have to take peanut butter off of that. That’s our challenge.”

## 4. Discussion

Despite the increasing prevalence of childhood obesity, community-based programs remain scarce and are warranted (Summerbell et al., 2003). Given that other research-based summer camps aimed at attenuating obesity rates have generally been residential in nature (Gately, Cooke, Butterly, Mackreth, & Carroll, 2000; Gately et al., 2005; Wong et al., 2009), C.H.A.M.P. represents a unique approach to the treatment of childhood obesity delivered as a summer day-camp. Residential programs can be costly, which is a significant deterrent for many lower-income families (Wong et al., 2009). Social and economic factors play an influential role in shaping health and disease patterns (USDHHS, 2010). In line with this tenet, we were able to offer C.H.A.M.P. to families at a low cost due to significant external funding. Parents expressed that the affordable fee, in comparison to the high cost of many other typical summer day-camps, facilitated their decision to enroll. Through C.H.A.M.P., all families had equal access to the same resources, professionals, guest speakers, and activities, irrespective of their socio-economic backgrounds. In light of the positive findings gleaned through the present study, the day-camp format may be a worthwhile consideration for prospective programs that are designed to increase accessibility to quality childhood obesity treatment.

An overwhelming theme across all of the focus groups was a perceived increase in self-esteem and self-efficacy among the children, a finding which supports previous research exploring parental perceptions of treatment for childhood obesity (Stewart, Chapple, Hughes, Poustie, & Reilly, 2008). For some parents, this improvement was identified as more important than a successful weight outcome, and attributed primarily to the judgement-free C.H.A.M.P. culture of positivity, as well as the social support and encouragement that were provided by the other participants and program staff. This is an interesting finding given that significant changes to body composition variables did occur amongst the children following the 4-week program (e.g., decreased body fat percentages, waist circumference, and weight; Shapiro et al., under review), yet the positive enhancements to psychosocial well-being were more readily discussed across all of the focus groups. For example, parental proxy reports pertaining to the physical, emotional, and social quality of life of the children showed significant improvements over the course of the 4-week intervention (Shapiro et al., under review). Based on the viewpoints of these parents, and in accordance with research conducted by Dixey and colleagues (2006), it may be the case that addressing the emotional well-being of children with obesity is an important precursor to actual or perceived weight loss/body composition changes. Thus, it appears that avenues for enhancing and better understanding these constructs should be given priority when developing and implementing treatment programs.

In order to facilitate sustainability and buy-in, families were invited to attend bi-monthly “booster sessions” for one year following completion of the 4-week day-camp. Across the focus groups, parents expressed that the weekend sessions held during C.H.A.M.P. were valuable, and many stated they were eager to meet again during the school year. However, attendance at the five booster sessions declined substantially following the formal intervention (2008: 60% - 29%; 2009: 67% - 17%), indicating that greater attention should be paid to adherence enhancing strategies during and after the treatment period. For example, it may be the case that a more directive approach is necessary. Although numerous tools and strategies were provided during the Saturday sessions, some parents still expressed a desire for more step-by-step, customized information. In addition to the group-based education sessions, working with the parents one-on-one or in smaller groups could allow for a greater focus on personal facilitators and barriers (e.g., time management, lack of buy-in from other family members), thus enabling the identification of personalized solutions. Moreover, this format could also be useful for enhancing accountability (e.g., regarding assignments, goal setting) which was a concern for some of the parents.

## 5. Conclusion

In light of the important role that early life events play in shaping an individual's health trajectory (USDHHS, 2010), this study provides important insights into the facilitators and barriers for healthy behaviour change among parents whose children are struggling with obesity. Based on these findings, in addition to those reported previously (Shapiro et al., under review), C.H.A.M.P. seems to provide a promising approach for childhood obesity treatment through its programmatic focus on positivity, social support, and self-esteem. However, one limitation to the present study is the fact that not all parents were able to attend the focus group discussions. Thus, it is possible that the views held by non-participating individuals were different from those who did participate. Further, because the focus groups were conducted by individuals known to the participants through their involvement in the program, it may have been the case that regardless of the honesty demands used, some responses were influenced by social desirability. These limitations should be considered for future behaviour-based research programs aimed at exploring parental viewpoints in this context. Additionally, in light of the present findings, intensifying the parent-focused component of family-based interventions targeting childhood obesity in service of promoting long-term adherence and sustainability of health behaviour changes is also a worthwhile consideration.
